# Uranium record from a 3 m snow pit at Dome Argus, East Antarctica

**DOI:** 10.1371/journal.pone.0206598

**Published:** 2018-10-31

**Authors:** Xiang Zou, Shugui Hou, Ke Liu, Jinhai Yu, Wangbin Zhang, Hongxi Pang, Rong Hua, Paul Mayewski

**Affiliations:** 1 School of Geographic and Oceanographic Sciences, Nanjing University, Nanjing, China; 2 CAS Center for Excellence in Tibetan Plateau Earth Sciences, Beijing, China; 3 Climate Change Institute, University of Maine, Orono, Maine, United States of America; 4 School of Earth and Climate Sciences, University of Maine, Orono, Maine, United States of America; Kaohsiung Medical University, TAIWAN

## Abstract

Understanding the distribution and transport of Uranium is important because it can lead to both chemical and radiological toxicity. This study presents the Uranium concentrations time series from 1964 to 2009 obtained from a 3 m deep snow pit at Dome Argus, East Antarctic Plateau. The data shows that Uranium concentrations vary from 0.0067 pg g^-1^ to 0.12 pg g^-1^, with a mean concentration of 0.044 pg g^-1^. Its mean concentration is 2–3 folds lower than at West Antarctica study sites, such as the Antarctic Peninsula (mean 0.12 pg g^-1^), IC-6 (Ice Core-6) (mean 0.11 pg g^-1^) and a suite of ice cores from the US ITASE traverse. Before the mid-1980s, the varieties of Uranium concentrations are relatively stable, with a very low mean concentration of 0.016 pg g^-1^and its main source is sea salt deposition, while a small number of anthropogenic sources are likely to be caused by Uranium mining operations in South Africa. A remarkable increase of Uranium concentrations has occurred since the mid-1980s (by a factor of ~ 9) compared with the amount before the mid-1980s. This increase coincides with the Uranium records at IC-6 and Antarctic Peninsula (DP-07-01) during the same period, and are mostly attributed to Uranium mining operations in Australia as a potential primary anthropogenic Uranium source. Our observations suggest that Uranium pollution in the atmosphere might have already become a global phenomenon.

## Introduction

Antarctica, with a unique geographical condition, can store large quantities of airborne ions and elements in snow and ice which can be used to investigate past environmental changes and atmospheric circulation patterns [1]. Dome Argus (Dome A) is located at the highest point of the Antarctic inland, at an altitude of 4093 m.a.s.l. (meters above sea level), about 1228 km from the nearest coastline and it is called “the inaccessible pole” as a result of the farthest distance from the coastline [2]. In Dome A, the average snow accumulation rate was 23 kg m^-2^ yr^-1^ and the mean surface temperature was -58.3 °C [2]. Trace elements stored in Dome A can act as climatic indicators to reveal the climate and environmental changes with global scale characteristics due to the scarce human interference.

**Fig 1:**
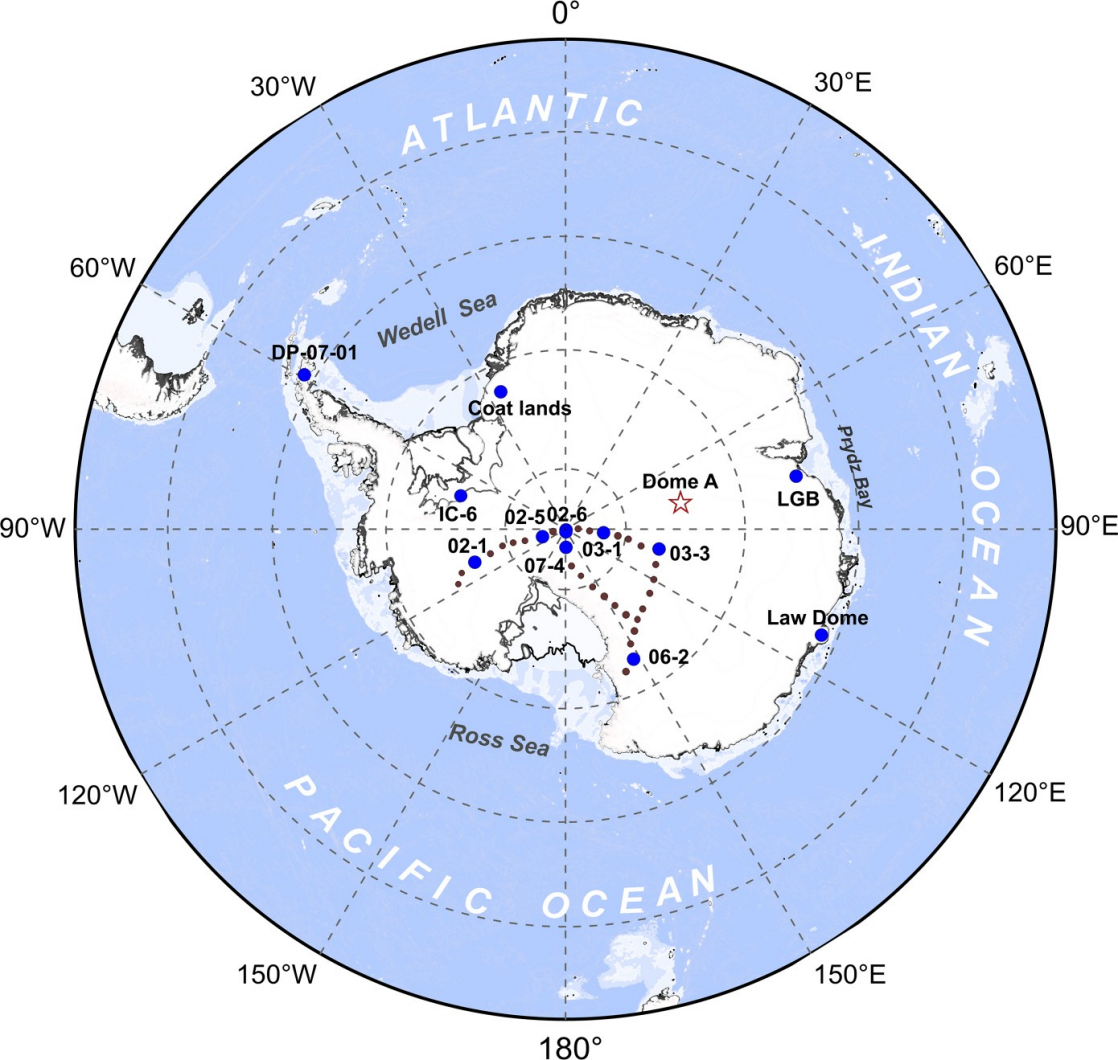
Map of Antarctica showing the site of the snow pit at Dome A (pentagram), as well as the sites of the previous studies about Uranium concentration, including DP-07-01 [15], Coat lands [16], IC-6 [17], LGB [18], Law Dome [19] and a series of snow pits along the US ITASE traverses (shown by brown dots) [20]. The topographic data were extracted using ETOPO1 elevations global data, available from National Oceanic and Atmospheric Administration at http://www.ngdc.noaa.gov/mgg/global/global.html (Last access: 4 October 2018).

Uranium is a rare and natural radioactive element with an average abundance of 1.7 ppm in the upper crust [3, 4]. Uranium can enter the body from the respiratory tract in the form of dust and aerosols and cause kidney damage because of its chemical radiotoxicity [5]. However, there is no reference concentration to assess the risk of inhaling exposed Uranium. Uranium is also a vital metal element for the development of human civilization and the exploitation of nuclear industry. Due to World War Ⅱ, the Uranium concentrations exposed to the air have increased significantly all over the world [6]. There have been numerous studies on the Uranium environmental pollution, such as marine Uranium radiation pollution [7], salt marsh Uranium pollution [8], groundwater [9], river systems [10], lakes [11], soils [12], plants [13], and atmosphere [14], but there has been few researches on Uranium pollution in snow and ice, especially in Antarctica. According to our understanding, the Uranium records were only reported in Antarctic Peninsula (DP-07-01) [15], Coats Land [16], IC-6 (Ice Core-6) [17], Law Dome [18], LGB (Lambert Glacier Basin) [19] and a suite of ice cores from the US ITASE traverse [20] (Fig 1). These studies of Antarctic Peninsula, Coats Land, IC-6 have shown an increasing trend of anthropogenic influences on Uranium deposition in the recent decades, to a large extent caused by Uranium mining operations in the Southern Hemisphere [15, 16, 20]. However, the studies of Law Dome and LGB have shown that Uranium concentrations were mainly affected by natural sources, such as sea salt deposition, volcanic emissions [19]. Thus, the study of Uranium source in Antarctica is significant to access Uranium transport of air masses caused by human interference under the hemispheric scale.

This study presents the data coming from 26th Chinese Antarctica Expedition on the changes of Uranium concentrations over the last ~50 years (1964–2009) at Dome A. A total of 30 samples were analyzed for ions and trace elements. Combined with the historical compiled inventories of Uranium production and our data, we analyzed the temporal and spatial distribution characteristics, possible sources and transport mechanisms of Uranium in Antarctica.

## Methods

### Sampling

In this study, a 3 m depth snow pit was excavated near Dome A (80°22'S, 77°21'E). The samples were obtained in January 2010 during the 26th Chinese National Antarctica Research Expedition. The State Oceanic Administration granted permission for the fieldwork. Strict precautions were taken to prevent potential sampling contamination. A continuous series of 30 snow samples were collected at 10cm intervals by pushing a ultraclean PTFE (Poly tetra fluoroethylene) bottle into the wall of the snow pit. Then the bottles were packed in acid-cleaned LDPE bags and kept frozen until analysis. All bottles and sampling implements followed the strictly acid-cleaned procedure [21] to avoid potential contamination before use. In order to date the snow pit, a total of 20 samples from the bottom of snow pit 2–3 m at 0.05 m intervals for the beta activity measurement were collected.

### Analytical procedures

The trace elements and major ions were determined in the Climate Change Institute (CCI), University of Maine in Orono, USA. All samples were placed in ultraclean room (Class 1000) for melting at room temperature (~20°C). Samples (2.5 mL) for trace-element analysis were transferred to an acid-cleaned (Optima HNO_3_) vial and were acidified to 1% with HNO_3_ (Fisher ‘Optima’ Grade) under an ultraclean room (Class 100). The 30 samples were digested for two days. Trace elements were measured by a Thermo ELEMENT2 high resolution Inductively Coupled Plasma Sector Field Mass Spectrometer (ICP-SFMS). ICP-SFMS is equipped with an Apex inlet sample system to prevent sample contamination and improve machine sensitivity. Before analysis, the SLRS-4 frozen river water reference material for trace metals (National Research Council Canada, Ottawa, Canada) was used for adjusting ICP-SFMS [22]. Trace elements were measured in a high resolution and a sensitivity of ^115^In = 800 000 cps per 100 ng L^-1^. The Uranium method detection limit was 6.46 fg g^-1^, defined as three times the standard deviation of three process blanks. Samples (4 mL) for major ions (Ca^2+^, Mg^2+^, Na^+^, K^+^, NH^4+^, NO^3-^ and Cl^-^) analysis were transferred to another vial. Major ions were analyzed by ion chromatography (IC) equipped with DX-500 chromatographs fitted with suppressed conductivity detectors and Gilson autosamplers. The AS-11 column, 400 μL sample loop and the eluent of 5 mM KOH was selected for anion measurement. The CS-12A column, the 500 μL sample loop and the eluent of 25 mM methanesulfonic acid were selected for cation measurement.

The samples for *β*-activity measurement were melted at the room temperature in ultraclean room (Class 1000). In order to fully activate the radioactive material in the samples, hydrochloric acid (38%, ‘Huafu’, Yangzhou, China) was added to the sample at a level of 0.00033 mL kg^-1^. Afterwards, the acidified samples were filtered on a cation exchange filter membrane (MN616 LSA-50, Germany) and a anion exchange filter membrane (MN616 LSB-50, Germany) for three times. The prepared filter membranes were analyzed for *β*-activity in the SKLCS (State Key Laboratory of Cryospheric Sciences, Chinese Academy of Sciences, Lanzhou, China) by using a MINI 20 Alpha-Beta Multidetector (Eurisys Mesures, St. Quentin, France).

### Snow pit dating

**Fig 2:**
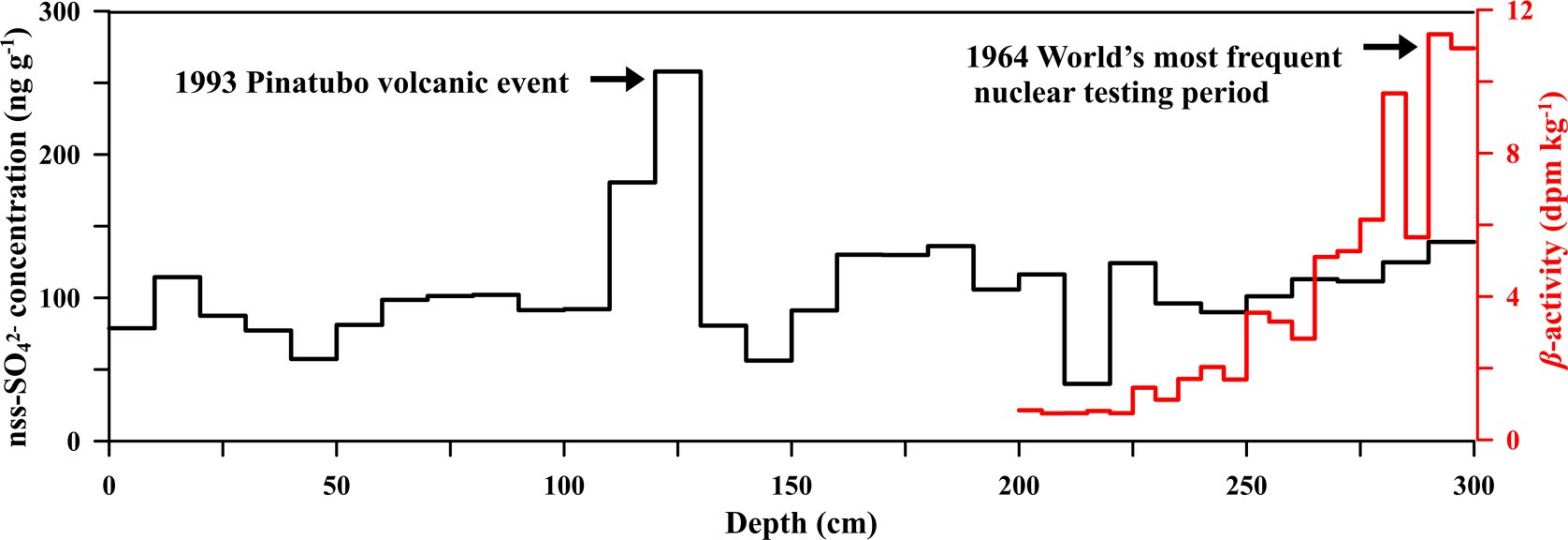
Profiles of the non-sea salt sulfur (nss-SO_4_^2-^) concentration (black solid line) and the *β*-activity (red solid line) of the snow pit.

The world’s most frequent atmospheric nuclear tests occurred during the period 1962–1963, which released a great deal of radioactive material resulting in a radioactive horizon of Antarctic snow in 1964–1965 because of a 1.5 yr transporting time [23, 24]. In this study, *β*-activity peak with a value of 11.32 dpm kg^-1^ at the depth of 2.9 m (Fig 2), corresponding to the 1964–1965. Non sea salt sulfur (nss-SO_4_^2-^) is a proxy for reflecting volcanic events, which is calculated using the following equation:

$${\mathrm{nss}‑\mathrm{SO}}_{4}^{2‑}={\mathrm{SO}}_{4}^{2‑}‑0.25{\mathrm{Na}}^{+}.$$ (1)

The nss-SO_4_^2-^ peaked with a value of 257 ng g^-1^ at the depth of 1.3 m (Fig 2), might be caused by the volcano eruptions in Pinatubo in 1991. According to Cole-Dai et al [25], the sulfur signals of Pinatubo recorded in South Pole snow and ice last from the late 1992 to the late 1993, since each sample in this study covers a period 1–1.5 yr, thus, we corresponded 1993 to the boundary of the nss-SO_4_^2-^ peak (Fig 2). Depth-year series were calculated based on the settled marked layers and the density of each sample. The calculated average snow accumulation rate was 18.2 kg m^-2^ a^-1^ from 1965 to 1993 and 22.4 kg m^-2^ a^-1^ from 1993 to 2009, respectively, which was consistent with former observations in Dome A [2, 26]. Our dating results was supported by previous studies [2, 26, 27].

## Results and discussion

### Results

**Fig 3:**
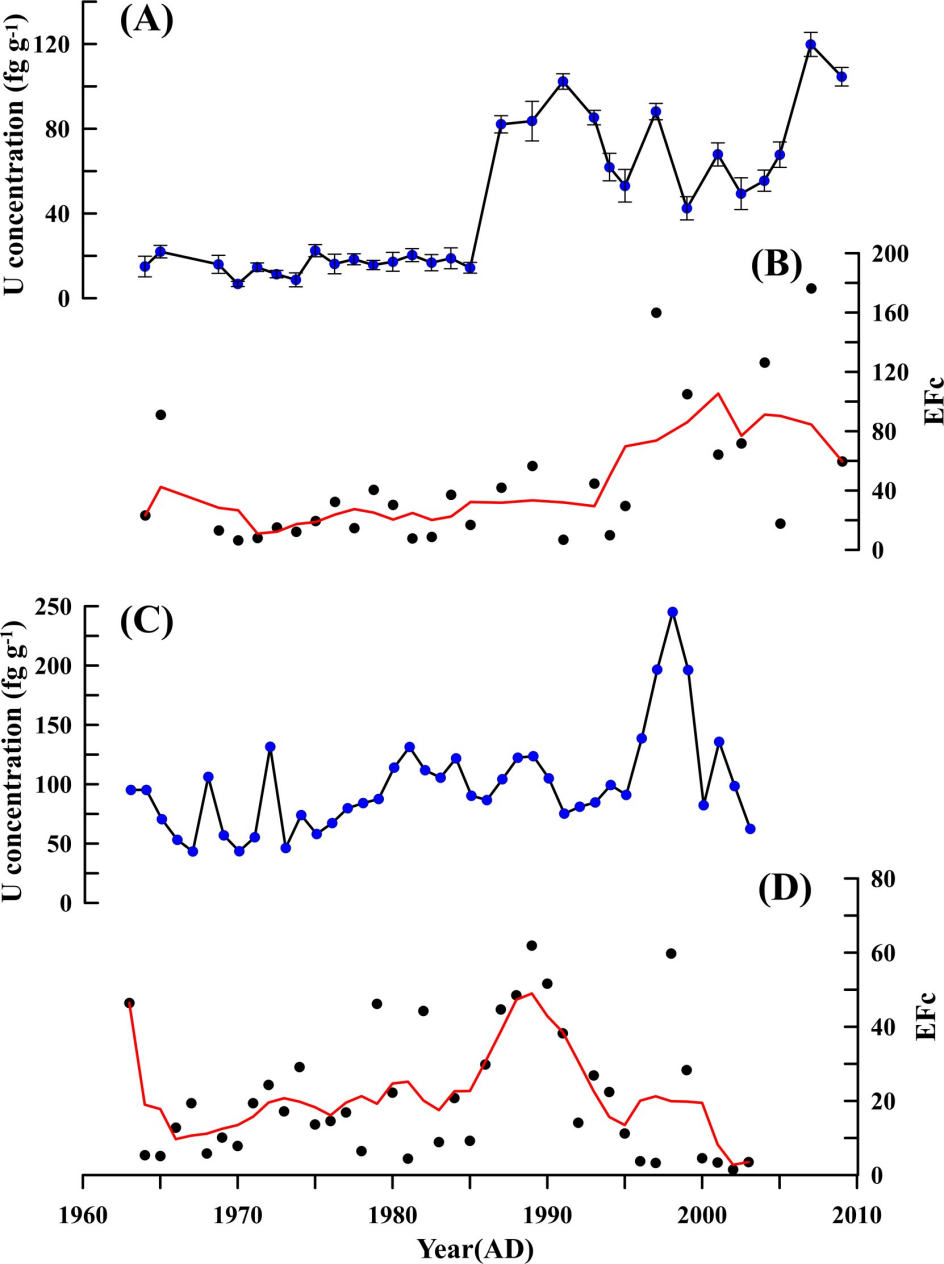
Profiles of Uranium concentrations and EFc. (A) Uranium concentrations at Dome A. (B) EFc of Uranium at Dome A. (C) Uranium concentrations at IC-6. (D) EFc of Uranium at IC-6 (solid red lines represent five-year running averages).The Uranium concentrations data at IC-6 was obtained from Carlos (2012)[17].

Fig 3A shows the changes of Uranium concentrations from 1964 to 2009. Uranium concentrations vary from 0.0067 pg g^-1^ to 0.12 pg g^-1^, with a mean of 0.044 pg g^-1^ after blank correction. From 1964 to the mid-1980s, Uranium concentrations were low, ranging from 0.0067 pg g^-1^ to 0.0224 pg g^-1^, with a mean of 0.016 pg g^-1^. In the early-1990s, Uranium concentrations increased greatly, reaching 0.082 pg g^-1^. Then the Uranium concentrations began to drop from the early 1990s to the early 2000s, subsequently rise and reach a maximum of 0.12 pg g^-1^ at the end of the 2000s. Uranium concentrations in this study are comparable with other study sites in Antarctica. More information is presented in Table 1.

**Table 1:** Uranium concentration ranges and mean values at Dome A and other Antarctic sites.

Location	Time period (A.D.)	Elevation(m)	U conc (pg g^-1^) (mean)	Ref
Dome A (80°22′S,77°21′E)	1964–2009	4093	0.0067–0.12 (0.044)	This work
Coats Land (SiteA:77°34′S,25°22′W) (SiteB:77°15′S,18°05′W)	1919–1990	1400	0.011–0.079 (0.037)	[16]
IC-6 (81°03′S,79°50′ W)	1934–2002	750	0.00177–0.6 (0.11)	[17]
Antarctic Peninsula (64°05′S,59°39′W)	1980–2007	1900	0.023–0.5 (0.12)	[15]
LGB (70°05′S, 77°04′ E)	1998–2002	1850	0.014–0.078 (0.029)	[19]
Law Dome (66°46′S, 112°48′E)	4500 BC- 1989	1390	0.009–0.042 (0.03)	[18]
ITASE-02-1 (82°00′S,110°00′W)	1966–1975	1746	(0.153)^a^	[20]
ITASE-02-5 (88°00′S,107°98′W)	1967–1975	2747	(0.168)^a^	[20]
ITASE-02-6 (89°93′S,144°39′E)	1955–1975	2808	(0.069)^a^	[20]
ITASE-03-1 (86°84′S,95°31′E)	1955–1975	3124	(0.094)^a^	[20]
ITASE-03-3 (82°08′S,101°96′E)	1955–1966	3444	(0.119)^a^	[20]
ITASE-06-2 (77°78′S,152°37′E)	2002–2006	2277	(0.137)^a^	[20]
ITASE-07-4 (88°50′S,178°53′E)	2000–2006	3090	(0.062)^a^	[20]
ITASE-07-5 (89°78′S,171°43′E)	2000–2006	2808	(0.050)^a^	[20]

^a^ Mean value only

### Natural contributions

The natural sources of trace elements in the atmosphere mainly include the upper crust, volcanic eruption and sea salt spray [28]. In this study, the crustal enrichment factor (EFc) has been used for assessing the contribution of upper crust to Uranium deposition at Dome A. We estimate EFc according to the following formula:

$$\mathrm{EFc}=\frac{{\left[\frac{U}{r}\right]}_{\mathrm{sample}}}{[{\frac{U}{r}]}_{\mathrm{upper}\;\mathrm{crust}}}$$ (2)

where r refers to the reference element [29]. Here, Titanium (Ti) has been used as a reference element to evaluate the crustal contribution [15]. Generally, the upper crust is considered to be the main source if EFc varies from 0.1 to 10, while EFc over 10 indicates additional sources [30]. Fig 3B presents the EFc of Uranium, ranging from 6.46 to 176.25 with a mean of 44.89 during the period 1964–2009. Overall, EFc remains at a lower level, with only a few values greater than 10 before the mid-1980s, which suggests that there is only a small amount of external sources to Uranium deposition at Dome A during that period. EFc has increased significantly since the mid-1980s. The results coincide with the observations of Uranium studies in Antarctic Peninsula [15] and IC-6 (Fig 3D) [17]. These observations indicate that there has been a large number of additional sources affecting the Uranium deposition not only at Dome A but also at other study sites in Antarctica since the mid-1980s, which suggests the increases of Uranium concentrations at Dome A are not a regional phenomenon.

Apart from assessing the upper crust, we also analyzed the effect of sea salt spray on Uranium deposition at Dome A. Planchon et al [16] and Hur et al [19] thought that sea salt spray was an important source to Uranium deposition. This is largely attributed to their study sites proximity to the coast. We used sea salt sodium, sodium in the snow and elemental ratios in seawater to assess the sea salt contribution to Uranium deposition by the following formula [27, 31]:

$$Sea\;salt\;contribution=\frac{{\left[\frac{U}{\mathrm{Na}{‑\mathrm{Na}}_{\mathrm{sea}\;\mathrm{salt}}}\right]}_{\mathrm{sample}}}{{\left[\frac{U}{\mathrm{Na}}\right]}_{\mathrm{seawater}}}$$ (3)

**Fig 4:**
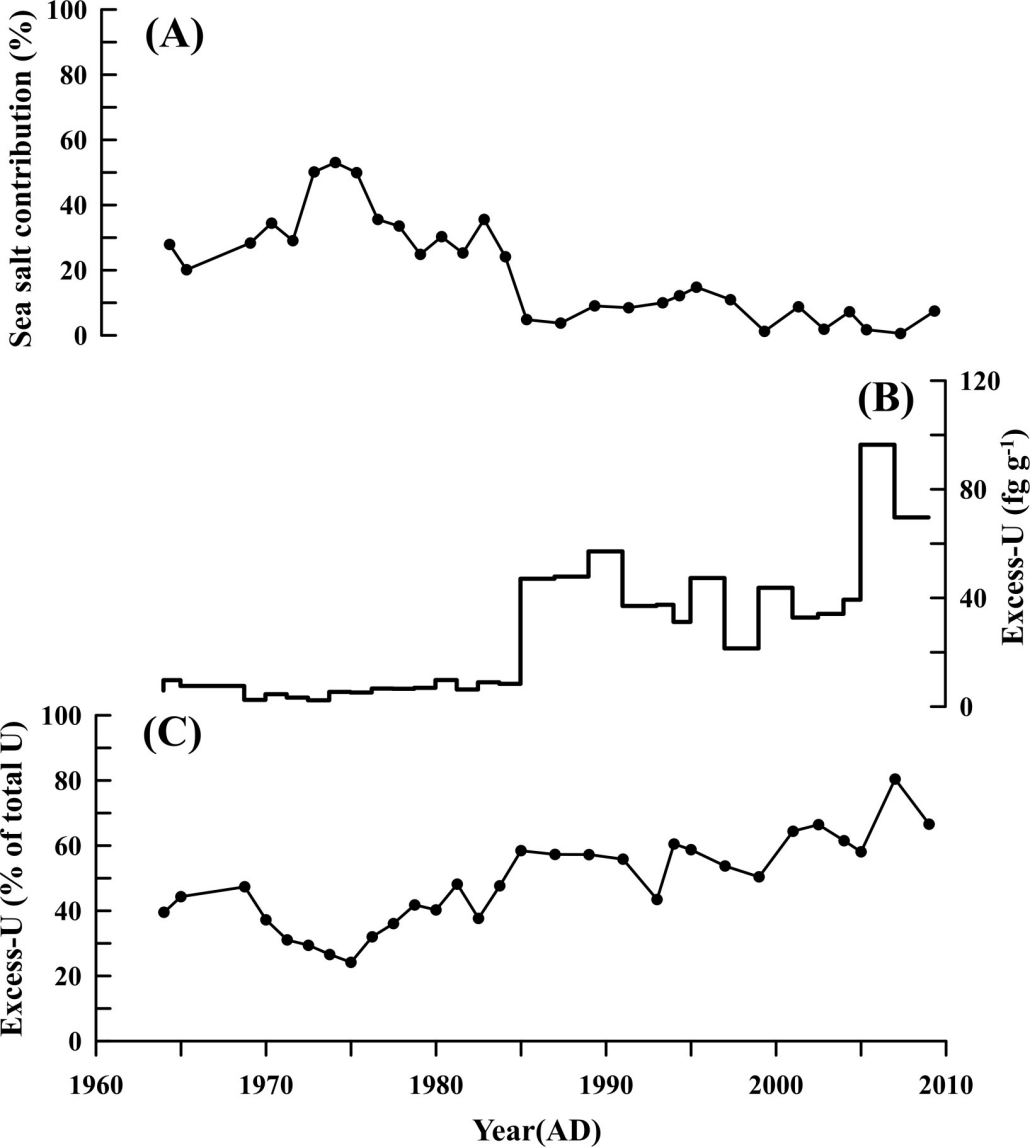
Sea salt contribution and Excess U. (A) Sea salt contribution to Uranium deposition. (B) Excess U. (C) Excess U accounts for % of total U.

The calculated sea salt contributions (Fig 4A) display the two changing periods. Before the mid-1980s, sea salt contribution was relatively high, with a mean of 33.5% and a maximum of 53.1%. After the mid-1980s, sea salt contribution dropped rapidly, with an average of 6.9% and a maximum of 14.8%. The results suggest that sea salt contribution has been weakening while anthropogenic contribution has played a vital role since the mid-1980s. To further distinguish the natural sources and anthropogenic sources of Uranium, excess Uranium (defined as xsU) was calculated using the following formula:

$$xsU=U_{\mathrm{total}}-U_{\mathrm{ocean}}-U_{\mathrm{crust}}$$ (4)

where U_ocean_ and U_crust_ refers to oceanic source and crustal source, respectively [15]. Generally, the anthropogenic sources are significant when xsU (% of total U) is greater than 60% [15]. As shown in Fig 4C, the results show that xsU (% of total U) is less than 60% before mid-1980s, indicating that the additional sources to Uranium deposition account for only a small part during that period. After the mid-1980s, xsU (% of total U) increased with the Uranium concentrations (Fig 3A), reaching a maximum (80%) at the late 2000s. The results of xsU and sea salt contribution suggest that the additional sources have played an important role for the Uranium deposition since the mid-1980s, while sea salt contribution decreased due to an increase of additional sources in Dome A.

Finally the volcanic contributions to Uranium deposition at Dome A were assessed by nss-SO_4_^2-^ (Fig 2). The peak of nss-SO_4_^2-^ in 1993 corresponds to the peak of Uranium concentrations, which has a maximum of 102.31ng g^-1^. This suggests that the volcanic event of Mt. Pinatubo (1991 A.D.) is likely to affect Uranium deposition at Dome A by atmospheric deposition.

### Anthropogenic contributions

The previous studies on the sources of heavy metals in Antarctica have shown that the reasons for the enrichments of heavy metals are possibly related to mining operations and mineral smelting in the Southern Hemisphere [15, 27, 29, 32]. According to Ohshima et al [33] and Barbante et al [6], Uranium mining and milling is a main source for Uranium concentrations in the atmosphere because such activities can result in large amounts of dust, with a significant amount of small particles which can be transported over long distances in the atmosphere. Thus, in order to assess the possible contribution by Uranium mining to Uranium deposition at Dome A, we compared our data with the historical records of the Southern Hemisphere Uranium mining operations, mainly including three major regions such as Australia, South Africa (only Zambia), South Africa (only Argentina and Brazil) (data from minerals UK center for sustainable mineral development at http://www.bgs.ac.uk/mineralsuk/statistics/wms.cfc?method=searchWMS).

**Fig 5:**
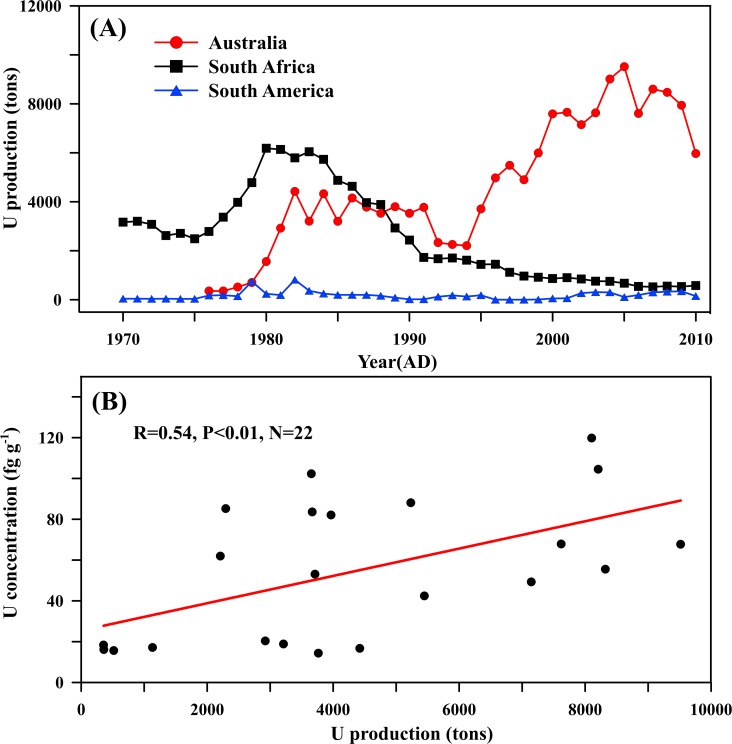
Uranium production and correlation. (A) The Uranium production of Australian, South Africa, South America from 1970 to 2010 (data from minerals UK at http://www.bgs.ac.uk/mineralsuk/statistics/wms.cfc?method=searchWMS). (B) The correlation between the annual U concentration and the annual production of Australia (T-test).

Fig 5 shows the changes of Uranium production from 1970–2010 in Australia, South Africa, and South America, respectively. The results suggest that the world’s Uranium production stabilized at a very low level before 1980, and just Uranium production in South Africa is largest, reaching to 2000 tons. Combined with the results of Uranium concentrations (Fig 3A) and EFc (Fig 3B), a small number of additional sources to Uranium deposition at Dome A before the mid-1980s were possibly related to Uranium mining operations in South Africa because the dust from South Africa is also an important source for heavy metal deposition in Antarctica [34]. Subsequently, the Uranium productions in South Africa has been descending whereas Australia has become the largest supplier of Uranium in the Southern Hemisphere since 1980. Comparing the Uranium production in Australia with our data, we observed that there is a strong correlation between the Uranium production in the Southern Hemisphere and Uranium concentrations at Dome A, both of which have risen consistently since the 1980s. The correlation coefficient between Uranium concentrations and Australian Uranium production was 0.54 at the 99% confidence interval (R = 0.54, P<0.01, N = 22) (Fig 5B). The interannual variability in Australian Uranium production agrees well with the corresponding the variabilities of Uranium concentrations at Dome A, which suggests Uranium mining operations in Australia as potential primary anthropogenic Uranium sources. The similar observations can also be found in the previous literature [15]. In sum, our results show that the anthropogenic sources to Uranium deposition at Dome A can be attributed to Uranium mining operations in the Southern Hemisphere especially in Australia, which indicates Uranium pollution in the atmosphere might have already become a global phenomenon by atmospheric circulation.

### Transport

**Fig 6:**
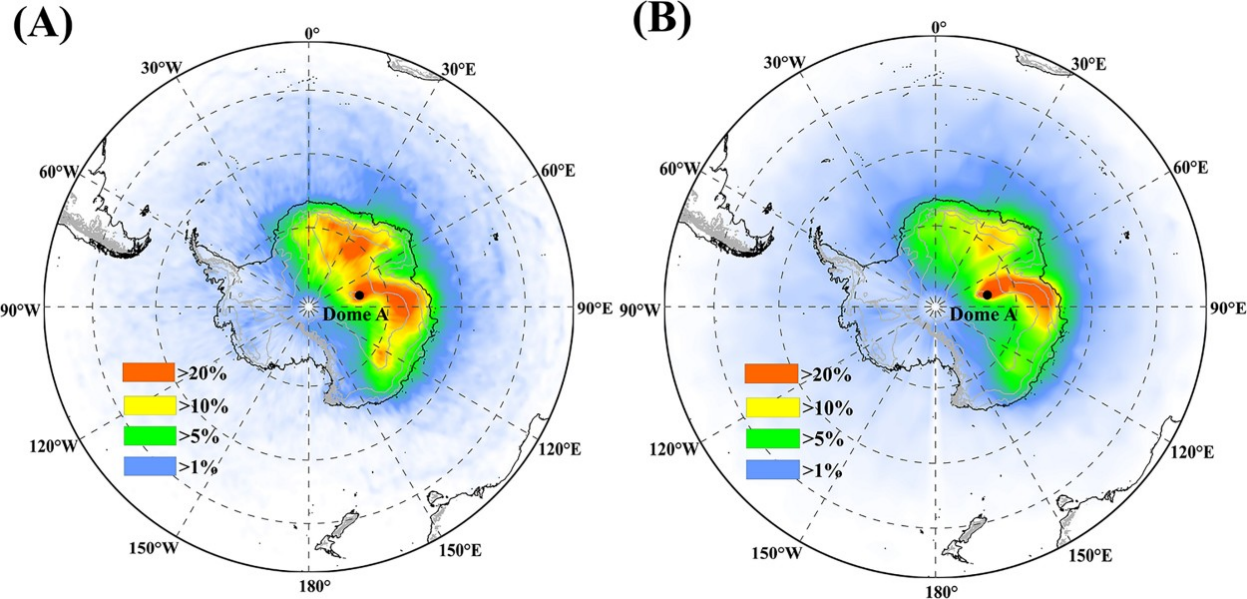
**Frequency plot of 15-day back trajectories at 500 m (A) and 1000 m (B) height levels above the ground representing the airflow trajectories at the middle and high altitudes respectively for daily simulation during the period 2000–2009**. The Back trajectory data obtained using the NOAA (National Oceanic and Atmospheric Administration, USA) HYSPLIT model (at ftp://arlftp.arlhq.noaa.gov/pub/archives/reanalysis).

In order to trace the potential transport path of air masses, we analyzed the 15-day back trajectories in Dome A, using HYSPLIT (Hybrid Single-Particle Lagrangian Integrated Trajectory) model. HYSPLIT is a complete system for calculating simple air masses trajectories and complex transport, distribution, and deposition simulations, developed by the National Oceanic and Atmospheric Administration (NOAA)’s Air Resources Laboratory (ARL) [35]. In this study, the air masses backward trajectories at 500m height (Fig 6A) and 1000m height (Fig 6B), respectively for daily simulations from 1 January 2000 to 31 December 2009, a period of high Uranium production in Australia, are used in Dome A. As shown in Fig 6, there are two major sources of air masses to Dome A from the northeast and the northwest respectively at 500m height (Fig 6A), but only one major source of air masses to Dome A come from the northeast when it is set at 1000m height (Fig 6B). It is known that westerly winds caused by pole-equator temperature and pressure gradients in the Southern Hemisphere are located between ~30°S and ~60°S, centered at approximately 50°S [36, 37]. Westerly winds play a key role in transporting the dust resulting from the mid-latitudes of Australia, New Zealand, South America and South Africa to the Southern Ocean and Antarctica [38]. According to a 10-day forward trajectories modeling of modern dust transported to the Southern Ocean and Antarctica [34], the dust from Australia can be transported to West Antarctica or further by westerly winds. Thus, the air masses from the northwest (Fig 6A) in Dome A were possibly affected by westerly winds and eventually precipitated at Dome A.

The Southern Annular Mode (SAM) is a vital mode of climate variability which can dominate the westerly winds at mid- to high-latitudes in the Southern Hemisphere [39]. According to Laluraj et al [40], a great deal of dust flux to East Antarctica was observed since the mid-1980s, coinciding with a shift in the SAM index to positive phase. This phenomenon is interpreted as the positive phase of SAM index that can strengthen westerly winds which increases the amount of dust transported to Antarctica [27]. Thus, the increases of Uranium concentrations at Dome A have been also likely caused by the positive phase of SAM index since the mid-1980s.

To further ascertain air mass transport in Antarctica and verify the reliability of our data, we compared our data with Uranium concentrations at IC-6 because IC-6 is near Dome A and its Uranium concentrations time series from 1934–2003 coincide with the period in Dome A. The result shows mean Uranium concentration (0.099 pg g^-1^) (Fig 3C) at IC-6 during the period 1964–2003 is twice as high as that at Dome A (Fig 3A) during the period 1964–2009. There are two possible reasons. One is that the average snow accumulation rate at IC-6 is 300 kg m^-2^ a^-1^ [17], which is approximately 10 folds higher than that at Dome A (23 kg m^-2^ a^-1^) [2], resulting in a great deal of air masses precipitation at IC-6. The other is that IC-6 is located in West Antarctica, one facing the Pacific Ocean where it receives much more dust from Australia compared to East Antarctica [34]. As shown in Fig 3A and Fig 3C, the variabilities of Uranium concentrations at IC-6 are similar to that at Dome A after the mid-1980s, suggesting that Uranium pollution caused by Uranium mining operations in Southern Hemisphere, especially Australia, was not a regional phenomenon in Antarctica.

## Conclusions

This study presents Uranium concentrations time series from 1964 to 2009 at a depth of 3m in Dome A, East Antarctica. Before the mid-1980s, the Uranium concentrations in Dome A are greatly affected by sea salt deposition and a small number of additional sources are possibly caused by Uranium mining operations in South Africa during that period. After the mid-1980s, Uranium concentrations have significantly increased, which coincide with the study of Uranium record at IC-6 and Antarctic Peninsula (DP-07-01) at the same period. This phenomenon has been largely attributed to many Uranium mining operations in Australia since the mid-1980s. The results indicate that Uranium pollution might has become a global phenomenon dispersal by atmospheric circulation.
